# Educating during the COVID-19 Pandemic: An Educator Perspective on Mental Health

**DOI:** 10.1017/dmp.2026.10334

**Published:** 2026-03-24

**Authors:** Alyssa Schneider Carlson, Zoe Sirotiak, Stephanie Orellana, Emily B. K. Thomas

**Affiliations:** Department of Psychological and Brain Sciences, The University of Iowa, United States

**Keywords:** COVID-19, qualitative, educators, pandemics, school

## Abstract

**Objective:**

During the COVID-19 pandemic, educators were impacted across domains of psychological, physical, social, and occupational health. Occupational environments, including classrooms, changed significantly due to COVID-19. This study aimed to characterize the nuanced experience of educators during the COVID-19 pandemic.

**Methods:**

Participants included 918 United States educators (e.g., teachers, paraeducators, support professionals, and administrators). Educators completed the survey via Qualtrics during November and December of 2020. The survey included eight qualitative questions, and responses were randomized to inductive or deductive datasets for analyses.

**Results:**

Our deductive results showed that individuals reported changes in several facets of health, with educators reporting increases in stress (38.0%) and feelings of isolation (45.9%). Our results also showed impacted occupational health, citing increased responsibility at work (34.0%) and feeling as though their voice was not heard after voicing concerns to either building- or district-level administration (28.2%).

**Conclusions:**

One of the key findings that emerged at the systemic level was educators reporting a lack of decision-making agency within the workplace. Through including educators in the conversations regarding decisions at all levels, agency may be increased. Educators not only shape the students they teach, but the communities within which they work, making a focus on their health paramount.

## Background

The COVID-19 pandemic has adversely impacted global health.^1^ COVID-19 infections caused widespread mortality, with over 7 million deaths worldwide.^2^ Symptoms of COVID-19 infection include fever, cough, shortness of breath, fatigue, headache, pain, sore throat, congestion, nausea, and diarrhea.^3^ Physical symptoms may remain significant and impairing following infection, including fatigue, reduced exercise capacity, and decreased ability to perform physical roles.^4^ ^,^^5^ The pandemic has impacted mental health, characterized by increased psychological distress,^6^ as well as psychiatric symptoms, such as depression, anxiety, and posttraumatic stress disorder.^6^ ^–^^9^ Downstream effects of the COVID-19 pandemic, including social isolation, financial stressors, health risk, and lifestyle changes, also impacted mental health.^10^ ^–^^12^

The occupational environment has also changed significantly across multiple industries. Workers experienced significant changes, including working from home, face covering implementation, physical barrier assembly, and improved ventilation.^13^ These changes have been associated with a decrease in life satisfaction.^14^ Factors associated with occupational environmental stressors include fear of infection or infecting others, job strain, periods of quarantine or isolation, perception of worker rights exploitation, and uncertainty.^15^

The mental health impacts of the COVID-19 pandemic have not been uniform across occupational groups. Essential workers, defined as those whose jobs necessitated in-person work, reported significant fear, stress, anxiety, depression, insomnia, burnout, and distress.^16^ ^–^^18^ In addition to being more likely to become infected and die from COVID-19,^19^ ^,^^20^ essential workers faced other adversities, such as a lack of personal protective equipment, staff shortages, challenges with enforcing regulations, and delays.^17^ ^,^^19^ ^,^^21^ ^,^^22^ Moreover, when lockdowns were enforced in the USA, testing was largely available to symptomatic or exposed individuals, contributing to stress about asymptomatic spread among essential workers.^23^ Essential health care workers reported high levels of fear, anxiety, depression, and trauma related to the pandemic, leading to burnout and feeling unable to ensure patient safety.^24^ A qualitative study performed with health care workers identified inadequate preparedness, emotional challenges, insufficient equipment and information, and work burnout as related to the pandemic.^24^

Educators are a group of essential workers who were significantly impacted throughout the COVID-19 pandemic. Educators are defined as teachers, administrators, paraeducators, and support professionals. Research has demonstrated that teachers reported high rates of anxiety, depression, stress, and burnout related to the COVID-19 pandemic.^25^ ^–^^28^ Many educators were expected to continue teaching, and the struggles of USA educators were outlined in an American Psychological Association (APA) technical report,^29^ which indicated that many educators desired to transfer or quit education, received verbal threats of violence, and experienced physical aggression from students. A related policy brief emphasized that investigating psychological symptoms among educators has never been more timely.^30^

Factors that contribute to the development of psychological distress and disorder have been identified among educators. Lack of collegial support, fear of verbal or physical abuse, significant workload, student behavior, and poor employment conditions were associated with depression, anxiety, and poor mental health.^31^ ^–^^33^ Among USA educators, increased stressors during the COVID-19 pandemic were related to poorer mental health, and poor mental health was associated with increased difficulty coping and teaching.^34^ Additionally, most Wisconsin, USA, educators reported clinically significant depressive and anxiety symptoms.^35^ Moreover, schoolteachers in Australia reported worsened mental health, citing contributors including increased responsibilities, changes in ways of working, and difficult working conditions.^16^ In Poland, educators reported at least mild levels of stress, anxiety, and depression, noting contributing factors including blurred professional and personal boundaries and isolation.^36^ The transition from in-person to remote teaching contributed to poorer mental health, with remote teaching associated with higher levels of distress than in-person^37^; however, other work has demonstrated high depressive and anxiety symptoms that did not differ based on teaching modality.^38^ Increased workload due to lockdown was suggested as a potential cause of worsened educator mental health.^39^ Uncertainty, increased workload, negative perception of the profession, concern for the well-being of others, health struggles, and juggling multiple roles were related to poor mental health among educators in England.^40^

Although quantitative research offers distinct advantages, qualitative and mixed methods research provides nuanced perspectives and context regarding individual experiences of collective events. Specifically, qualitative research methods can clarify the lived experience of individuals.^41^ Qualitative research may be particularly helpful in characterizing the lived experience of mental health difficulties.^42^ COVID-19 and the mental health challenges that it poses may be particularly conducive to qualitative investigation.^43^ Even though educators were among the essential workers faced with significant changes and dangers related to COVID-19, qualitative research investigating the effects of the COVID-19 pandemic on educators is limited. Given that many individual and societal experiences secondary to COVID-19 are novel, qualitative investigations are critical to provide contextual nuance. The APA policy brief^30^ and technical brief^29^ included both qualitative and quantitative data, further emphasizing the importance of both methods to capture educator experiences more fully during the COVID-19 pandemic. In response to the APA reports and the gap in the current literature, this study aims to understand how the COVID-19 pandemic has impacted mental, physical, occupational, and social health among educators.

## Method

### Participants

Participants (n = 918) were English-reading/speaking adults in the USA who identified as an educator (e.g., teacher, paraeducator, administrator) from kindergarten through college. The average age of the sample was 43 years (*SD* = 11.3). Most identified as White (96.6%) and female (87.8%). At least one person participated from 46 states of the USA. To be included in the qualitative analyses, a response to one of the 8 qualitative questions was required. There were 145 participants who were excluded due to lack of qualitative response, leaving a sample of 925. Participant responses were randomized into 2 samples for analyses: inductive and deductive. See the demographic characteristics of the two samples in Table 1.

**Table 1.** Descriptive characteristics of the inductive(n = 224) and deductive samples (n = 701)

	Inductive, n (%)	Deductive, n (%)
Age, *M* (SD)	43.12 (10.6)	43.13 (11.4)
Personal Income^1^		
$0 through $49,999	74 (33.0%)	227 (32.5%)
$50,000 through $74,999	110 (49.1%)	344 (49.1%)
$75,000 and greater	31 (13.8%)	102 (14.6%)
Unknown	0 (0%)	5 (0.7%)
Missing	9 (4.0%)	23 (3.3%)
Race		
White	214 (95.5%)	681 (97.1%)
Asian	1 (0.4%)	3 (0.4%)
Biracial or Multiracial	7 (3.1%)	10 (1.4%)
Native Hawaiian or Pacific Islander	1 (0.4%)	0 (0%)
African American or Black	0 (0%)	3 (0.4%)
American Indian or Alaska Native	0 (0%)	3 (0.4%)
Did not disclose	1 (0.4%)	1 (0.1%)
Ethnicity		
Non-Hispanic	213 (95.1%)	678 (96.7%)
Hispanic	6 (2.7%)	14 (2.0%)
Did not disclose	5 (2.2%)	9 (1.3%)
Gender Identity		
Female	196 (87.5%)	630 (89.9%)
Male	24 (10.7%)	67 (9.6%)
Transgender man	4 (1.8%)	0 (0%)
Genderqueer/gender non-conforming	0 (0%)	2 (0.3%)
Prefer to self-describe	0 (0%)	1 (0.1%)
Prefer not to disclose	0 (0%)	1 (0.1%)
Role in school system		
Administrator	14 (6.3%)	53 (7.6%)
Teacher	202 (90.2%)	625 (89.2%)
Paraeducator	8 (3.6%)	23 (3.3%)

### Procedures

Potential participants clicked a link to the survey, which directed them to the survey at qualtrics.com. Congruent with the institutional review board (IRB)-approved protocol, the first page of the survey was a consent letter notifying participants that clicking to the next page indicated consent to participate. The consent specified that participation was voluntary, that the data would be stored on a secure computer drive, and that information provided by participants would not be identifiable. This protocol was approved by the University of Iowa IRB, approval #202007406. Both recruitment and data collection procedures are further detailed alongside quantitative findings.^38^ The qualitative questions that were included are listed below.

1. How has your role as an educator during the pandemic impacted your ability to do your job day-to-day?
2. How has your role as an educator during the pandemic impacted your professional advancement and career goals?
3. How has your role as an educator during the pandemic impacted your mental health?
4. How has your role as an educator during the pandemic impacted your physical health?
5. How has your role as an educator during the pandemic impacted your social health?
6. Do you feel that your school community was receptive and/or responsive to your preferences for educating during the COVID-19 pandemic?
7. Were your preferences solicited by a school board, administrative body, county governance, or state governance, and if so, did these preferences appear to be considered in decision-making processes?
8. Please feel free to speak to the degree to which you felt your voice as an educator was valued during the pandemic.

### Data Analysis

The dataset was divided into an inductive and deductive cohort, using random assignment with a 1:3 scheme (inductive: deductive). In the inductive phase, randomly selected participants were utilized for coding. The purpose of this phase was to develop codes, identify themes, and identify representative data to support codes and themes.^44^ The deductive phase utilized the inductive codes for deductive coding of the data. The purpose was to allow for a structured, systemic analysis to enhance the validity of codes and themes.^45^ Four coders were involved in analyses. Each coder reviewed all responses to qualitative questions in the inductive dataset. A thematic discussion resulted in the development of relevant codes. Responses to each question were reviewed sequentially to ensure independent consideration for thematic extraction.

The data analytic plan and hypotheses were pre-registered through Open Science Framework (OSF) prior to randomization of responses.^46^

### Thematic Analysis and Codebook Formation

The themes were formulated based on responses to the 8 qualitative questions, and codes were formed across questions. After identification of themes and the codebook, the inductive dataset was coded by all coders. All codes were reviewed iteratively for accuracy, clarity, and agreement.

#### Inductive data analyses

In code generation, coders noted themes of interest.^47^ Individual extractions were collated into themes, ensuring that all data relevant to a theme were included.^47^ Coders reviewed codes and themes for coherence, accuracy, and refinement.^47^

#### Deductive data analyses

Responses were randomly assigned to coders. The deductive codes are reported for interpretation. In addition to random assignment, 30% of each coders’ data were randomly selected for double coding to ensure agreement between coders. All discrepancies were resolved through discussion until agreement was reached.^45^ ^,^^48^

## Results

The results demonstrate that this sample reported declines in physical, mental, and social health, as well as challenges in their professional environment. Educators reported not feeling that their voices were being heard regarding implemented workplace policies. Educators reported taking precautionary measures personally and professionally to prevent infection or spread of COVID-19.

When individuals were recorded as having “not reported” a code, this means that it was not mentioned explicitly. When a participant was recorded as having “reported” a code, this experience was explicitly mentioned and was available for coding. See Table 2 for theme frequencies in each phase.

**Table 2.** Frequencies of inductive and deductive codes

	Inductive, n (%)	Deductive, n (%)
Fear of COVID–19 infection	53 (24.0%)	82 (11.6%)
Fear of spreading COVID–19	51 (14.4%)	93 (13.1%)
COVID–19 exposure		
Exposed through work	9 (2.5%)	29 (4.1%)
Exposed to COVID–19, unspecified origin	4 (1.1%)	6 (0.8%)
COVID–19 infection		
Contracted COVID–19 at work	4 (1.1%)	4 (0.6%)
Infected with COVID–19, unspecified origin	5 (1.4%)	6 (0.8%)
Limited social interaction	271 (76.8%)	510 (72.0%)
Family time		
Less time with family	64 (18.1%)	106 (15.0%)
More time with family	2 (0.6%)	3 (0.4%)
Lack of connection or feeling isolated	65 (18.4%)	197 (27.8%)
Forgoing activities		
Choosing not to attend a general category of activities	176 (49.9%)	325 (45.9%)
Not attending specific events	9 (2.5%)	19 (2.7%)
Virtual activity (i.e., engaged in virtual activity, e.g., Zoom)	113 (32.0%)	211 (29.8%)
Exercise or physical activity		
Decreased	63 (17.8%)	116 (16.4%)
Increased	15 (4.2%)	35 (4.9%)
Increased sedentary behavior	43 (12.2%)	64 (9.0%)
Increased alcohol consumption	6 (1.7%)	5 (0.7%)
Increased self-care	16 (4.5%)	14 (2.0%)
Weight changes		
Weight gain	43 (12.2%)	88 (12.4%)
Weight loss	4 (1.1%)	17 (2.4%)
Diet changes		
Negative changes in diet	33 (9.3%)	69 (9.7%)
Positive changes in diet	1 (0.3%)	11 (1.6%)
Sleep changes		
Sleeping less	21 (5.9%)	27 (3.8%)
Sleeping more	3 (0.8%)	3 (0.4%)
Sleep change, unspecified direction	3 (0.8%)	9 (1.3%)
Somatic symptoms		
Exacerbation of prior somatic symptoms	4 (1.1%)	4 (0.6%)
New onset somatic symptoms	34 (9.6%)	84 (11.9%)
Fatigue		
Exhaustion	42 (11.9%)	66 (9.3%)
Tiredness or fatigue	23 (6.5%)	69 (9.7%)
Exacerbation of physical illness	6 (1.7%)	22 (3.1%)
Lack of access to health behavior facilitators	14 (4.0%)	20 (2.8%)
Chronically ill	8 (2.3%)	15 (2.1%)
Anxiety	76 (21.5%)	148 (20.9%)
Stress	122 (34.6%)	269 (38.0%)
Depression	38 (10.8%)	93 (13.1%)
Grief/nostalgia	18 (5.1%)	34 (4.8%)
Guilt	15 (4.2%)	21 (3.0%)
Uncertainty	58 (16.4%)	91 (12.9%)
Frustration	72 (20.4%)	170 (24.0%)
Post-traumatic symptoms	5 (1.4%)	10 (1.4%)
Feeling imprisoned	8 (2.3%)	23 (3.2%)
Suicidality	5 (1.4%)	4 (0.6%)
Exacerbation of mental health difficulties	15 (4.2%)	20 (2.8%)
New onset of mental health difficulties	76 (21.5%)	169 (23.9%)
Medications		
Increased medication	4 (1.1%)	6 (0.8%)
Started new medication	10 (2.8%)	13 (1.8%)
Therapy		
New pandemic therapy	4 (1.1%)	7 (1.0%)
Current therapy, unclear if existent prior to pandemic	5 (1.4%)	11 (1.6%)
Desire to start but unable to access therapy	3 (0.8%)	4 (0.6%)
Helpless or hopeless	38 (10.8%)	131 (18.5%)
Lack of time for self	4 (1.1%)	19 (2.7%)
Alone	49 (13.9%)	153 (21.6%)
Solitude appreciation	5 (1.4%)	13 (1.8%)
Self-reflection	35 (9.9%)	30 (4.2%)
Falling short of professional self-expectations	49 (13.9%)	113 (16.0%)
Inadequate education for students	45 (12.7%)	124 (17.5%)
Overworked	42 (11.9%)	124 (17.5%)
Increased responsibility	84 (23.8%)	245 (34.6%)
Nature of job changed	43 (12.2%)	149 (21.0%)
Positivity	34 (9.6%)	16 (2.3%)
Unsafe policies or spaces	47 (13.3%)	163 (23.0%)
Student issues	14 (4.0%)	50 (7.1%)
Parental disrespect	15 (4.2%)	18 (2.5%)
Resentment towards profession	72 (20.4%)	191 (27.0%)
Financial concerns	27 (7.6%)	25 (3.5%)
Professional instability	16 (4.5%)	36 (5.1%)
District-level administrative support		
Less support	65 (18.4%)	142 (20.1%)
More support	5 (1.4%)	29 (4.1%)
Building-level administrative support		
Less support	17 (4.8%)	28 (4.0%)
More support	24 (6.8%)	39 (5.5%)
Administrative support, level unspecified		
Less support	46 (13.0%)	109 (15.4%)
More support	15 (4.2%)	19 (2.7%)
Voiced concerns		
Voiced concerns, not heard	91 (25.8%)	200 (28.2%)
Voiced concerns, heard	30 (8.5%)	70 (9.9%)
Told what they would be doing, not asked	59 (16.7%)	71 (10.0%)
Told what they would be doing, not asked *and* voice not heard	9 (2.5%)	25 (3.5%)
State influence on decision-making	30 (8.5%)	80 (11.3%)
Parental influence on decision-making	18 (5.1%)	41 (5.8%)
Other organizational influence on decision-making		
School board	32 (9.1%)	60 (8.5%)
Union	22 (6.2%)	22 (3.1%)
Family-work responsibilities	4 (1.1%)	16 (2.3%)
Work satisfaction		
Dissatisfaction with work	41 (11.6%)	97 (13.7%)
Satisfaction with work	10 (2.8%)	14 (2.0%)
Career change		
Desire to shift careers	39 (11.0%)	78 (11.0%)
Plan to shift careers	9 (2.5%)	7 (1.0%)
Shifted careers due to pandemic	7 (2.0%)	6 (0.8%)
Pursuing early retirement	12 (3.4%)	18 (2.5%)
Retired early	0 (0.0%)	2 (0.3%)
Educational pursuits		
Declined to pursue additional education	36 (10.2%)	41 (5.8%)
Pursued additional education	11 (3.1%)	34 (4.8%)
Desire to pursue additional education but no action reported	5 (1.4%)	10 (1.4%)
Career advancement		
No opportunity for career advancement	22 (6.2%)	9 (1.3%)
Experienced career advancement	4 (1.1%)	39 (5.5%)
Technology	20 (5.7%)	76 (10.7%)
Lack of resources, internet	3 (0.8%)	3 (0.4%)
Lack of resources, cleaning supplies	4 (1.1%)	2 (0.3%)
Lack of resources, personal protective equipment (PPE)	4 (1.1%)	13 (1.8%)
Parental academic support		
Less support	15 (4.2%)	10 (1.4%)
More support	9 (2.5%)	1 (0.1%)
Personal protective behaviors, seeking increased air flow	37 (10.5%)	51 (7.2%)
Personal protective behaviors, disinfecting surfaces	72 (20.4%)	131 (18.5%)
Personal protective behaviors, social distancing	93 (26.3%)	122 (17.2%)
Personal protective behaviors, requesting students to mask	37 (10.5%)	41 (5.8%)
Personal protective behaviors, personal PPE use	63 (17.8%)	138 (19.5%)
Personal protective behaviors, parental safety support	1 (0.3%)	3 (0.4%)
Personal protective behaviors, altering eating behaviors	148 (41.9%)	314 (44.4%)
Personal protective behaviors, altering shopping behaviors	80 (22.7%)	130 (18.4%)
Personal protective behaviors, increased cleaning of self	44 (12.5%)	90 (12.7%)
Personal protective behaviors, travel		
Limiting travel	10 (2.8%)	15 (2.1%)
No travel	12 (3.4%)	27 (3.8%)
Personal protective behaviors, isolating	65 (18.4%)	226 (31.9%)
Personal protective behaviors, altering living arrangements	8 (2.3%)	5 (0.7%)

*Note.* Data represent the frequency (*n*) and percentage (%) of participants in the inductive (*n* = 224) and deductive (*n* = 701) samples endorsing each code. Definitions for abbreviated or specific codes are as follows: *Alone* indicates feeling alone or uncared for; *Job change* indicates feeling as though the nature of the job has changed (e.g., feeling like a babysitter); *Positivity* indicates having to stay positive for the kids or behaving incongruent with emotions; *Student issues* indicates an increase in student behavioral issues; *Technology* indicates new technology as a factor; and *Unsafe policies or spaces* indicates policies or physical spaces do not feel safe at work or are not following CDC guidelines.

### Themes

*COVID-19.* Codes observed included: fear of COVID-19 infection, fear of spreading COVID-19, COVID-19 exposure, and COVID-19 infection. Exposure was defined by coders as a known exposure, and infection as a positive test.

*Social health.* Given the numerous changes in public health recommendations that may have necessitated changes in social behaviors, these themes included responses such as social distancing, lockdowns, isolation, or minimization of contact with others. Codes in this section included limited social interaction, increase or decrease in family time, forgoing activities, virtual activities, and lack of connection or feeling isolated.

*Physical health.* Derived codes included changes in exercise or physical activity, weight, and/or diet changes, sleep changes, somatic symptoms, fatigue, and lack of access to health behavior facilitators (e.g., gyms or health care visits). Because of the regulations that varied by state, some of these activities were forbidden or strongly recommended against. Others reported forgoing activities to minimize risk.

*Mental health.* Derived codes included psychological experiences, including depression, anxiety, suicidality, and helplessness. This section highlights changes to existing psychiatric medications or starting new psychiatric medication. Themes of seeking new psychotherapeutic services or desire but inability to seek therapy were observed. This section included stress, grief, uncertainty, self-reflection, and appreciation of solitude.

*Professional well-being.* Derived codes included a sense that the education being provided was inadequate. A theme of having unsafe policies or physical spaces, such as classrooms being unfit for social distancing guidelines, was observed. Themes regarding student issues, parental disrespect, financial concerns, and either the presence or lack of administrative support were observed. Educators reported feeling overworked and having increased responsibilities. Educators reported insufficient personal protective equipment (PPE) to safely work in-person. Themes of career dissatisfaction and plans for career change were present. Some reported having shifted careers. On the other hand, some reported feeling validated in their career choice and had an increased sense of belonging. Another theme was having one’s voice heard by administrators; some reported having a role in decision-making processes, whereas others voiced opinions and were ignored. Others were not asked for feedback and were informed of plans.

*Precautions taken.* Derived codes highlighted the precautions that educators were taking to avoid becoming infected with or spreading COVID-19. Themes included disinfecting surfaces, social distancing, and altering eating and shopping behaviors.

### Deductive Findings

Participants reported fear of spreading (13.1%) or becoming infected with (11.6%) COVID-19, with some reporting a known exposure or infection at work (0.6%). When connected with CDC county-level data, most participants who also completed the quantitative data portion of this study (88.0%) reported living in an area with a “high” rate of COVID-19 transmission.^38^ Most reported limiting social interactions (72.0%), and 27.8% reported feeling lack of connection or isolation. Moreover, 49.5% chose not to attend certain activities, and 29.8% reported virtual engagement in activities. Changes in mental and physical health were also reported. New-onset symptoms were reported, with 11.9% of participants reporting new-onset somatic symptoms and 23.9% reporting new-onset mental health symptoms. Less often noted were exacerbations of physical illness (3.1%) or mental health difficulties (2.8%). Participants reported decreased physical activity (16.4%), weight gain (12.4%), and negative changes in diet (9.7%). Others reported positive health behavior changes (e.g., positive changes in diet [1.6%], increased self-care [2.0%], and increased exercise or physical activity [4.9%]).

Alongside mental health difficulties, many specific emotions and symptoms were reported, including anxiety (20.9%), stress (38.0%), frustration (12.9%), and uncertainty (12.9%). Many reported feeling helpless or hopeless (18.5%) and alone (21.6%). Several personal protective behaviors were reported, including altering eating behavior (44.4%) and personal PPE use (19.5%). Many reported a lack of administrative support across district-level (20.1%), building-level (4.0%), and unspecified levels of administrative support (15.4%). Others reported administrative support (district-level: 4.1%; building-level: 5.5%). Many reported their concerns were not heard when voiced (28.2%), while others reported feeling heard (9.9%). Others were told of plans, not asked (10.0%). Some reported being told of plans, not asked for their concerns, *and* their voice was not heard (3.5%).

Underscoring the competing demands that educators faced, 34% reported increased work responsibility, and 21% reported that the nature of their job changed. Some participants felt overworked (17.5%), and others reported that expectations had increased in a way that was unachievable (16.0%). Some participants reported feeling that they were delivering an inadequate education, and almost one-quarter reported unsafe policies or spaces at work (23.0%). Some participants reported work dissatisfaction (13.7%), and participants reported feeling work-related resentment (27.0%).

## Discussion

The purpose of this study was to understand how the COVID-19 pandemic impacted domains of health (mental, physical, and social), day-to-day functioning, and career satisfaction among educators in the USA. Although research detailed the physical and psychological health impacts of COVID-19 on educators worldwide, limited work has focused on educators’ lived experiences. The present study sought to derive themes inductively and apply the derived codes to the deductive dataset. The authors describe educator experiences across domains of health as detailed by educators in November and December of 2020.

The findings demonstrated deleterious mental and physical health impacts, where participants reported feeling demoralized and distressed. Participants endorsed occupational changes, including planning or desiring to leave the profession, in part due to new responsibilities and insufficient time. Educators reported increased expenses for work supplies, such as cleaning supplies and PPE. Many educators reported isolation in service of protecting others, with some living separately from family or not seeing relatives, limiting access to supports. See Table 3 for deidentified quotes sampled from educators in the deductive phase of analyses.

**Table 3.** Deidentified quotes from participants in the deductive phase, grouped by themes

Mental health	Physical health	Social health	COVID-19	Professional well-being	Precautions taken
“I have constant anxiety and high stress. I had to go on antidepressants and sleeping pills.”	“Stress has wreaked havoc on my body, and I have gained 20 + pounds since March.”	“I don’t see anyone in person anymore.”	“I am anxious and worried about getting covid.”	“I am exhausted everyday at work. My workload has severely increased and I feel like I never have enough time to plan or grade or give feedback.”	“Eating in my own classroom, sleep in separate room than [partner] ([they] work with Covid + patients), cancelled all travel, avoid coworkers that dont wear their mask appropriately.”
“I started counseling and started taking Zoloft to handle stress and anxiety due to work and the pandemic.”	“It has caused me to exercise more. I have found that running is a very positive thing for me to do when I am stressed out.”	“I miss spending time with friends and family. My mother has dementia [and] my father is taking care of her alone.”	“I have asthma and allergies and I’m constantly worried about regular asthma/allergy symptoms vs covid symptoms.”	“I want a new career and don’t know what else to do. I’ve been a teacher for [many] years and I want to get out.”	“No— it seemed pointless given the amount of close contact already involved in my work day.”
“Drained it. I’ve never been so depressed and I don’t recall feeling helpless ever. Now, both daily.”	“I have had panic attacks, chest pains, trouble sleeping. I’m diabetic and my eating habits have declined due to stress, causing my sugars to spike. My sleep habits have also been disrupted.”	“In some ways, the pandemic brought me closer to my colleagues. People outside of my profession do not understand the amount of pressure on teachers nor the amount of time and energy we are spending trying to balance in-person instruction, virtual instruction, and protecting our health. So I am losing contact with non-educators.”	“I am potentially exposed to Covid every work day. I will not risk others.”	“I will leave education as soon as I have the opportunity to do so. My community and my leaders have abandoned any pretense of concern or respect for my profession or for my humanity.”	“Eating in my car, leaving work as soon as possible, never eat in restaurants, haven’t seen my family since March, haven’t seen most of my friends since March, using grocery pickup instead of going to the store.”
“I started seeing my therapist again starting in August due to the stress of work. My stress level is so high that I’ve just given up on being a good teacher.”	“Increased issues with blood sugar and hypertension as a result of the increased stress.”	“I stay away from my parents because they are high risk and I don’t want to catch something at work and then give it to them.”	“I acquired covid at work and am still suffering symptoms more than a month later.”	“I have a strong administration that really values teachers and I feel like my voice has been heard and that my voice is encouraged.”	“I wear scrubs to work. I take my shoes off outside before coming into my house where I immediately take a shower then wash my clothes from the day. Then I spray lysol on door knobs and my shoes and my work bag. I do all this before I even hug my kids and greet my husband.”
“I feel I am happier overall…I love teaching online now”	“Weight gain, no activity, drinking more alcohol due to stress.”	“I can no longer see my family (who were the only people I’ve visited in their house the entire pandemic). I live alone and am now isolated.”	“I believe I had covid but didn’t realize it at the time and spread it to my family. Everyone in my family became ill. My mother in law ended up in hospital.”	“I was not given an option to teach remotely, despite having a heart condition and despite having elderly individuals in the household. Even now, I will soon be forced to choose FMLA.”	“Wearing scrubs not using public restrooms. Not eating in restaurants. Not seeing family or friends. Not attending meetings [or] getting haircut, massage.”

*Note.* Quotes that include [bracketed text] were altered to remove any potentially identifying information.

Our results align with literature noting worsened physical and mental health during the COVID-19 pandemic among educators worldwide.^6^ ^–^^9^ ^,^^49^ Decreased physical activity, increased sedentary behavior, weight gain, negative dietary changes, and sleeping less were noted among our sample, fitting with literature noting a decrease in healthy lifestyle behaviors during the COVID-19 pandemic.^49^ ^,^^50^ Somatic symptom burden was also significant, fitting with literature noting increased somatic symptom burden during the COVID-19 pandemic.^51^ Anxiety, stress, depression, uncertainty, and feeling hopeless or helpless were reported in our sample, aligning with extant research.^6^ ^–^^9^ ^,^^49^ Some participants noted increased self-reflection, fitting with research observing improved self-concept, autonomy, and psychological well-being among some during COVID-19 lockdowns.^52^ As with a significant proportion of the world’s population,^11^ our sample reported limiting social interaction and forgoing activities. Although concerns about developing or spreading COVID-19 were frequently noted among our sample, confirmed exposure and infection were relatively low, though infection was not directly assessed herein.

Past literature has noted educators as essential workers affected by the pandemic with reports of lack of colleague support, increased workload, and poor employment conditions.^25^ ^,^^29^ ^,^^31^ ^,^^32^ ^,^^53^ ^–^^55^ Our results further illustrate the lived experience of educators during this time. The participants noted increased responsibility, with failure to meet professional self-expectations, perception of providing inadequate education, being overworked, and feeling that the nature of the job had changed. These reports mirror wider literature noting increased responsibilities, juggling multiple roles, and the blurring of professional-personal boundaries.^16^ ^,^^36^ ^,^^39^ ^,^^40^ ^,^^55^ The APA technical report highlighted concerns regarding the safety of educators regarding physical violence, verbal abuse, and risk of COVID-19 infection.^29^ The present findings describe unsafe policies and physical spaces at work related to the infectious nature of COVID-19, further highlighting educator distress.

Educators noted varied experiences regarding support in the workplace. Building-level administrative support was reported more frequently than district-level administrative support, indicating that those in closer proximity to our sample (primarily teachers) provided more support. Several participants voiced concerns to superiors about the occupational environment with varying levels of success. The highest proportion who voiced concerns did not feel those concerns were heard. The perception of difficult working conditions has been associated with mental health difficulties among educators,^16^ ^,^^31^ ^,^^32^ ^,^^56^ and occupation-related concerns and lack of administrative support noted among some in our sample mirror this finding. Perhaps associated with these issues, resentment of employers was observed. Some educators noted desire or plan to shift away from educational careers, whereas others intended to pursue early retirement. These findings were also demonstrated by the APA, with 49% of US educators surveyed having a desire to leave the profession.^29^

Finally, pandemic precautions were reported during early stages of the COVID-19 pandemic,^57^ and our sample also endorsed protective behaviors. The most common protective behaviors were increasing ventilation, disinfecting surfaces, social distancing, using PPE, and altering shopping behaviors, fitting with findings in the wider population from this time.^58^ ^–^^60^ However, other precautions seemed specific to the occupational environment, such as monitoring masking among students and altering eating behaviors (e.g., not eating in classroom or break room).

### Limitations

There are several limitations to this research. The sample was primarily White, non-Hispanic females. Even though this aligns with the population of USA educators, our sample lacks diversity in race, ethnicity, and gender.^29^ ^,^^61^ The study advertisement was posted on Facebook and Reddit. This may have resulted in a lack of responses from educators who did not use social media. The study was cross-sectional; therefore, no longitudinal conclusions can be drawn. The data did not capture duration or intensity of experiences. Data were collected via survey to maximize participant ease, but no follow-up or clarifying questions were asked.

As our study involved educator responses during 2020, our findings do not apply to educators’ experiences during other phases of the pandemic. Longitudinal work could be utilized to assess the experience of educators over time. While our work analyzed written qualitative responses, other qualitative interviews or focus groups may allow follow-up and in-depth understanding of the educator experience. Future work could assess long-term implications of the COVID-19 pandemic on educators.

### Implications

Given the expressed mental health concerns, systemic emphasis on utilization of mental health care resources may be a helpful future direction. Providing resources in the workplace would improve access. One study in Colorado demonstrated workplace integration, with eight schools providing social-emotional competencies workshops in the workplace, and demonstrated benefit.^62^

Beyond individual-level interventions, systems-level interventions should also be considered. School psychologists are often confused as resources for the students and teachers, which is typically not the case. School psychologists serve as an invaluable resource for students struggling with issues related to learning and school, but oftentimes there are few (if any) resources for educator mental health within the school system.^63^ However, when providing a mental health care specialist for educators has been tested, mindfulness has improved, and stress has decreased.^64^ Although this research is promising, it does not address the needs of educators in areas that are not well-resourced. Districts that cannot provide an on-site mental health care specialist for educators should consider providing low-cost, virtual mental health care for employees.

The “Supporting the Mental Health of Educators and Staff Act of 2023” highlights the incorporation of mental health care services in the workplace and proposes substantial funding to support this type of programming at a policy level within the USA.^65^ Including mental health care screenings in employee health screenings would further the goal of normalizing checking in about mental health and well-being.^66^ Having referral options following screening is of critical importance.

At the systemic level, lack of agency or voice was described by educators in our sample. Many wrote about decisions being made on their behalf at the building and district levels. Encouraging representation of educators as key stakeholders in these choices would increase agency. Increasing spending for educator health would allow for identification of predictors of workplace satisfaction among educators.^67^ ^,^^68^ This would elucidate problem areas and improve teacher retention, leading to fiscal benefits and downstream improved student achievement.^67^ ^,^^68^

## Conclusion

In summary, the present study examined USA educators’ experiences. Many of the themes aligned with issues that have been highlighted in media and policy beyond the COVID-19 pandemic, including lack of perceived support by administration and inadequate time allotted for responsibilities. Our findings demonstrated negative impacts on educators’ physical and psychological health, as well as a desire to feel heard in decision-making. Further intervention research is needed to address educator mental and physical health at different levels of intervention (individuals, systems, policies). As educators shape the future of students and communities worldwide, it is imperative that their well-being is a top priority.

## Data accessibility

Deidentified or aggregated data will be made available upon reasonable request, congruent with the approved IRB application.
